# Improved motion correction using image registration based on variational synthetic image estimation: application to inline t1 mapping of myocardium

**DOI:** 10.1186/1532-429X-13-S1-P21

**Published:** 2011-02-02

**Authors:** Hui Xue, Saurabh Shah, Andreas Greiser, Christoph Guetter, Christophe Chefdhotel, Sven Zuehlsdorff, Jens Guerhing, Peter Kellman

**Affiliations:** 1 Siemens Corporate Research, Imaging and Visualization, Princeton, NJ, USA; 2 Siemens Medical Solutions, CMR Research and Development, Chicago, IL, USA; 3 Siemens AG, Healthcare Sector, Imaging & IT Division, Erlangen, Germany; 4 Laboratory of Cardiac Energetics, National Heart, Lung and Blood Institute, National Institutes of Health, Bethesda, MD, USA

## Introduction

**Figure 1:**
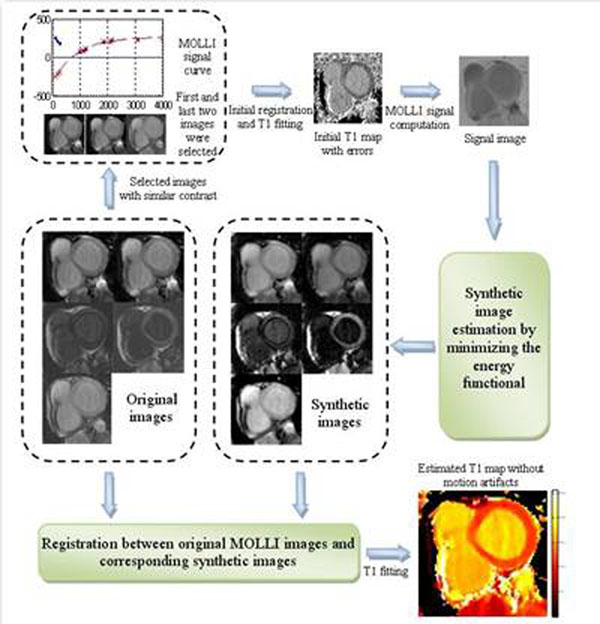
Synthetic image estimation based motion correction for T1 MOLLI series. Original images show a typical MOLLI series acquired across three heart-beats. 5 out of the total 11 images are plotted here. The estimated motion-free synthetic images show similar contrast to the corresponding original images where myocardial deformation is noticeable.

Myocardial T1 mapping has potential to quantify structural and pathological changes without use of contrast agent. Recently Modified Look-Locker Inversion Recovery (MOLLI) sequence has proven effective on cardiac T1 mapping [1]. Its clinical applicability is still limited by myocardial motion between frames mainly due to beat to beat variations and respiration. This undesired motion compromises the accuracy of pixel-by-pixel T1 estimation. Unfortunately, registration of MOLLI images is particularly difficult as image contrast changes significantly over time (Figure 1). A simple frame-to-frame registration often fails with unrealistic deformation. In this work, we propose a novel registration algorithm based on estimating motion-free synthetic images presenting similar contrast to original data by solving a variational energy minimization problem. Robust motion correction is achieved by registering synthetic images to corresponding MOLLI frames.

## Methods and materials

**Figure 2:**
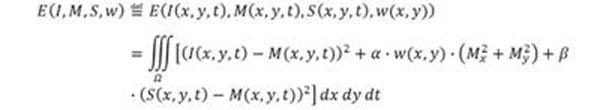
Definition of energy functional for the synthetic image estimation . Here ***I***(***x***, ***y***, ***t***) is the acquired MOLLI image, and ***S***(***x***, ***y***, ***t***) is the MOLLI signals which is calculated from the initial T1 parameter fitting. Note for the inversion recovery sequence, the polarity of acquired magnitude image has to be corrected. The weight function ***w***(***x***, ***y***) is added to maintain the edge sharpness in the estimated synthetic image. The first term constrains the distance between synthetic images and original MOLLI images. The second term is the regularizer using first-order derivative. It is added to penalize occasional error in the original T1 estimation and keep sufficient SNR of synthetic images. This term does not constrain the smoothness of the termporal behavior. The last term is added to minimize the distance between estimated images and the MOLLI signal recovery curve. As the MOLLI signal recovery curve is essentially smoothing, the term actually implicitly constrain the temporal smoothness of estimated synthetic images. ***α*** and ***β*** are weights to balance the contribution across three terms. This energy minimization problem can be solved by evaluating the resulting Euler equation.

4 volunteers and 9 patients were scanned (Siemens MAGNETOM Avanto/Espree/Verio, 42/18 pre/post-contrast series). Given *N* frames of MOLLI images with inversion time TI, synthetic image is defined as a function to minimize the energy functional defined in Figure 2. To estimate the initial signal image, few MOLLI frames with similar contrast are selected and an initial registration and T1 fitting is performed. Final registration is performed between each synthetic image and corresponding MOLLI frame. This process of estimation and registration is empirically iterated twice to correct for residual motion. A fast variational non-rigid registration algorithm [2] is applied here with localized cross correlation as the cost function. All processing steps require no user interaction and are fully integrated into the scanners’ reconstruction software. T1 maps are typically available in ~30s after the image acquisition.

## Validation and results

**Figure 3:**
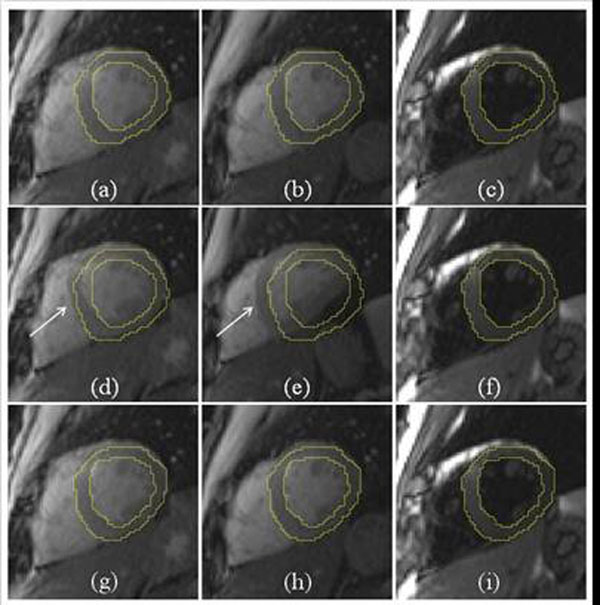
Example of MOLLI motion correction. (a-c) Original MOLLI images with contour overlay. Myocardium is still in this case. (d-f) Motion correction results by directly applying the non-rigid registration. Largely varying contrast causes the failure of registration, as shown in (d-e). (g-i) Motion correction based on synthetic image estimation.

**Figure 4:**
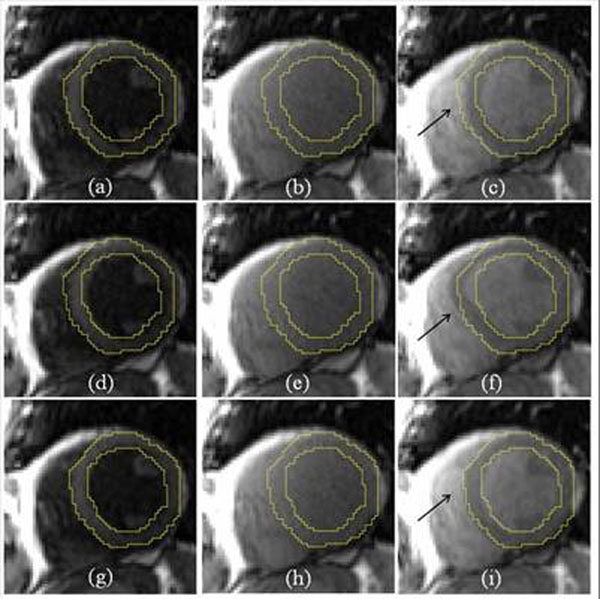
Example of MOLLI motion correction. (a-c) Original MOLLI images with contour overlay. Myocardial motion is noticeable in (c). (d-f) Motion correction results by applying the non-rigid registration directly between frames. Significant contrasts change failed the registration. (g-i) Motion correction based on synthetic image estimation.

Effectiveness was first evaluated by visual reading. All datasets were classified into two categories: 21 ‘no significant motion’ and 39 ‘with significant motion’. A frame-to-frame registration between images with largely varying contrast can lead to unrealistic deformation (Figure 3), which was found in 40 cases among the whole cohort (67%), while proposed approach was robust against contrast changes. Quantitative validation was performed on all cases with discernible motion (reconstructed in-plane resolution: 1.67 ~ 2.08 mm^2^). For every case, two frames with motion were selected and their myocardium was manually delineated. Two measures are computed (Table 1): *Dice ratio* and *MBE* (minimal distance between endo/epi contours of two frames). Typical performance is illustrated in Figure 4-5.

**Table 1:** The quantitative measures of motion correction based on synthetic images (MOCO).

	Dice ration		MBE (mm)	
	Original	MOCO	Original	MOCO
**Mean**	0.87	0.91	1.31	0.87
**STD**	0.07	0.05	1.35	1.06
